# Invasive pulmonary aspergillosis in critically ill patients with pneumonia due to COVID-19, influenza, and community-acquired pneumonia: A prospective observational study

**DOI:** 10.18502/cmm.8.2.10328

**Published:** 2022-06

**Authors:** Syed Ahsan Ali, Kausar Jabeen, Joveria Farooqi, Hammad Niamatullah, Aisha Fareed Siddiqui, Safia Awan, Alishah Akbar, Muhammad Irfan

**Affiliations:** 1 Department of Medicine, The Aga Khan University Hospital, Karachi, Pakistan; 2 Department of Pathology and Laboratory Medicine, The Aga Khan University Hospital, Karachi, Pakistan; 3 Aga Khan Medical College, Karachi, Pakistan

**Keywords:** *Aspergillus*, Aspergillosis, COVID-19, Influenza, Pneumonia, Outcomes

## Abstract

**Background and Purpose:**

Influenza A and SARS-CoV-2 are risk factors for invasive pulmonary aspergillosis. Both influenza-associated pulmonary aspergillosis and COVID-19-associated pulmonary aspergillosis result in high mortality and poor clinical outcomes.

No prospective study has so far compared the features, treatment, and outcomes of influenza-associated pulmonary aspergillosis and COVID-19-associated pulmonary aspergillosis within a similar time frame. Therefore, this study aimed to determine the frequency, risk factors, and outcomes of invasive pulmonary aspergillosis in critically ill patients with influenza, COVID-19, and community-acquired pneumonia.

**Materials and Methods:**

This prospective study included adult patients with pneumonia and was conducted at The Aga Khan University Hospital in Karachi, Pakistan. Patients were divided into three groups, including community-acquired pneumonia, influenza pneumonia, and COVID-19 pneumonia. The data collected included information on demographic characteristics, comorbidities, clinical features, laboratory results, treatment, and outcomes.

**Results:**

A total of 140 patients were included in this study. These included 35 (25%), 70 (50%), and 35 (25%) patients with community-acquired pneumonia, influenza pneumonia, and COVID-19 pneumonia, respectively. In addition, 20 (14.2%) patients were found to have invasive aspergillosis, of whom 10/35 (28.5%), 9/70 (12.8%), and 1/35 (2.8%) patients were in the COVID-19, influenza, and community-acquired pneumonia groups, respectively. Moreover, nine (90%) COVID-19-associated pulmonary aspergillosis patients required vasopressors, compared to three (33%) patients with influenza-associated pulmonary aspergillosis (*P=0.020*). In total, seven (70%) COVID-19-associated pulmonary aspergillosis patients required invasive mechanical ventilation compared to four (44%) influenza-associated pulmonary aspergillosis patients (*P=0.37*). The mean±SD length of hospital stay was highest in the COVID-19-associated pulmonary aspergillosis patients (18.3±7.28 days) compared to influenza-associated pulmonary aspergillosis patients (11.7±5.34 days) (*P=0.036*). The number of deaths in influenza-associated pulmonary aspergillosis and COVID-19-associated pulmonary aspergillosis patients was three (33.3%) and five (50%), respectively (*P=0.526*).

**Conclusion:**

A higher proportion of patients with COVID-19 developed invasive aspergillosis compared to those with influenza. Although the mortality rate in COVID-19-associated pulmonary aspergillosis was comparable to that in influenza-associated pulmonary aspergillosis patients, COVID-19-associated pulmonary aspergillosis patients had a significantly longer stay in the hospital.

## Introduction

Invasive pulmonary aspergillosis (IPA), once considered a disease of immunocompromised patients, has recently appeared in immune-competent patients. Several case reports have described IPA in immunocompetent but critically ill patients with risk factors, such as cirrhosis, chronic obstructive pulmonary disease, chronic alcohol abuse, burn injury, diabetes, malnutrition, and viral pneumonia [ 1 , 2 ]. Respiratory viruses, in particular influenza A and recently SARS-CoV-2, have been recognized as risk factors for the development of IPA [ 3 , 4 ]. Both influenza-associated pulmonary aspergillosis (IAPA) and COVID-19-associated pulmonary aspergillosis (CAPA) result in high mortality and poor clinical outcomes. Mortality and morbidity in CAPA are further compromised by azole-resistant organisms [ 5 , 6 ].

Although data on IAPA and CAPA have been reported from many countries, no prospective study so far has compared the frequency, risk factors, clinical and radiological features, treatment, and outcomes of IAPA and CAPA patients admitted within a similar time frame.

There is growing evidence that IPA develops in patients with viral pneumonia, especially those with influenza and COVID-19 pneumonia. However, no data have been available so far regarding the frequency of IPA in patients with community-acquired bacterial pneumonia (CAP).

This study aimed to determine the frequency, risk factors, and outcomes of IPA in patients with underlying influenza, COVID-19, and CAP admitted within a similar time frame.

## Materials and Methods

This prospective cross-sectional study was conducted at the high-dependency units (HDU) and intensive care units (ICU) of The Aga Khan University Hospital, Karachi, Pakistan from November 2019 to June 2020. 

### Sample Collection

Patients of age 18 years or more, of both genders, admitted to the HDU or ICU with a diagnosis of pneumonia were included in the study. Patients with hematological malignancy, absolute neutrophil count (ANC) of less than 1500/mm^3^, HIV, and those who had undergone organ transplantation were excluded from the study. Patients who were not willing to participate in the study were also excluded. The study was approved by the Ethics Review Committee (ERC) of The Aga Khan University (ERC # 2019-0847-2567). 

All patients with pneumonia and PCR positive for influenza on a nasopharyngeal swab or broncho-alveolar lavage (BAL) admitted from November 2019 to October 2020 were considered to have influenza pneumonia. Influenza virus was detected from nasopharyngeal swabs or bronchoalveolar lavage by real-time reverse transcriptase polymerase chain reaction (RT-PCR) using the Influenza virus PCR assay (Altona Diagnostics, GmbH). All patients admitted from April 2020 to October 2020 with pneumonia and positive nasopharyngeal or nasal swab for COVID-19 PCR were considered to have COVID-19 pneumonia. The SARS-CoV-2 virus was detected from nasal and nasopharyngeal swabs by RT-PCR and Cobas® SARS-CoV-2 Qualitative assay for use on the Cobas® 6800/8800 Systems (Roche Molecular Systems, New Jersey, USA). All other patients admitted with pneumonia, negative influenza, and COVID-19 PCR were considered to have community-acquired pneumonia.

*Aspergillus* species were identified using conventional techniques based on the colony morphology, color, texture, rate of growth, and microscopic morphology of phialides and conidiophores. Beta-D-glucan levels in serum were determined using Fungitell® (Associates of Cape Cod, Falmouth, MA, USA). In serum, a value of >80 pg/mL of Beta-D-glucan was considered positive. Galactomannan (GM) levels were determined using Platelia Aspergillus®, Bio-Rad Laboratories. In serum, a single positive index of >0.5 was considered positive. Similarly, in BAL, a single positive index of >1.0 was considered positive. 

CAPA was defined based on the definition provided by Kohler et al. and according to entry criterion, radiological, and mycological parameters [ 4 ]. IAPA was defined using the criteria mentioned by Verweij et al. [ 7 ]. IPA in CAP was defined based on the definition provided by Blot et al. [ 8 ]. 

The samples collected in the present study included sputum and tracheal aspirate samples. Patients who met the inclusion criteria were divided into three groups: community-acquired pneumonia, influenza pneumonia, and COVID-19 pneumonia. Demographic characteristics, level of care (HDU or ICU), comorbidities, clinical features, and laboratory results, including microbiological data, imaging, treatment received, complications, and outcomes were collected on a predesigned pro forma. 

For analysis of *Aspergillus*-positive patients, the comparison was made only between COVID-19 and influenza groups, and the patient with CAP was excluded from this analysis as this group had only one patient. Data analyses were conducted using a statistical package for social science (SPSS, version 19). A *P-value* less than 0.05 was considered statistically significant. An independent t-test or Mann-Whitney U test for non-parametric distribution was used to determine the p-values. A comparison was made for patients diagnosed with IPA among all three groups using the Chi-square test for categorical variables. For continuous variables, we used analysis of variance for parametric variables and the Kruskal-Wallis H test for non-parametric variables.

## Results

A total of 140 patients were included in the study. These included 35 patients (25%) with CAP, 70 patients (50%) with influenza pneumonia, and 35 patients (25%) with COVID-19 pneumonia. The demographic and clinical characteristics of all three groups of patients are presented in Supplementary Table 1. The mean±SD age of patients with CAP (65.9±12.31 years) was more than that in influenza (59.6±18.54 years) and COVID-19 (58.6±15.94 years) groups (*P=0.11*). A male predominance was observed in the COVID-19 group (91.4%) compared to influenza (45.7%) and CAP (60%) groups (*P<0.001*). The majority of the patients in the influenza group had influenza A H1N1 53 (75.7 %) while 15 (21.4 %) patients had non-H1N1 influenza A and two (2.9%) patients had influenza B.

Out of 140 patients, 20 (14.3%) developed possible IPA with CAPA observed in 10 out of 35 (28.6%), IAPA in 9 out of 70 (12.9 %), and IPA in CAP in 1 out of 35 (2.9%) patients. Differences in clinical characteristics, laboratory results, and radiological findings of patients with IAPA and CAPA are presented in Table 1. Treatment, complications, and outcomes of patients with IAPA and CAPA are presented in Table 2. Since only one patient developed IPA in the CAP group, it was not included in the analysis. 

**Table 1:** Clinical characteristics and investigations of Influenza and COVID-19 patients with invasive aspergillosis

	Influenza (n=9) (%)	COVID-19 (n=10) (%)	*P-value*
Age*(years)	65.44 (18.43)	59.7 (17.55)	0.497
**Gender**			
Male	4 (44.4)	8 (80)	0.109
Female	5 (55.6)	2 (20)	
**Level of Care**			
Intensive care unit (ICU)	5 (55.6)	7 (70)	0.515
High dependency unit (HDU)	4 (44.4)	3 (30)	
**Length of stay in Hospital** *	11.67 (5.34)	18.3 (7.29)	0.036
**Length of stay in ICU/HDU** *	9.4 (2.30)	17.0 (8.72)	0.034
**Co-morbidities**			
Diabetes mellitus	3 (33.3)	4 (40)	0.764
Hypertension	6 (66.7)	5 (50)	0.463
Ischemic heart disease	5 (55.6)	3 (30)	0.260
Chronic kidney disease	3 (33.3)	1 (10)	0.213
Stroke	0 (0)	2 (20)	0.156
Asthma	2 (22.2)	1 (10)	0.466
**Presenting Complaints**			
Fever	5 (55.6)	8 (80)	0.252
Shortness of breath	9 (100)	7 (70)	0.073
Cough	8 (88.9)	8 (80)	0.596
Hemoptysis	2 (22.2)	0 (0)	0.115
Disorientation	1 (11.1)	0 (0)	0.330
Gastrointestinal symptoms	2 (22.2)	0 (0)	0.115
**Duration of Symptoms** *	7.56 (4.00)	12.50 (12.13)	0.249
**Drug History**			
Glucocorticoids	1 (11.1)	1 (10)	0.737
Immunosuppressants	0 (0)	1 (10)	0.526
**GCS**			
<10	1 (11.1)	2 (20)	0.351
10-14	3 (33.3)	3 (30)	
15	5 (55.6)	5 (50)	
**APACHE II Score**			
0 - 24	4 (44.4)	8 (80)	0.060
25 - 34	1 (11.1)	2 (20)	
> 34	0 (0)	0 (0)	
**SOFA Score** *	9.4 (5.18)	5.4 (3.41)	0.170
**qSOFA Score**			
0-1	4 (44.4)	0 (0)
2-3	0 (0)	0 (0)
**Radiology findings**			
Infiltrates	5 (55.6)	10 (100)	0.011
Consolidation	5 (55.6)	10 (100)	0.001
Pleural effusion	4 (44.4)	2 (20)	0.259
Atelectasis	5 (55.6)	2 (20)	0.170
**Laterality of radiology findings**			
Bilateral	8 (88.9)	10 (100)	0.474
Unilateral	1 (11.1)	0 (0)	
**pH**			
Acidemia (<7.35)	0 (0)	3 (30)	0.175
Normal (7.35 - 7.45)	3 (33.3)	3 (30)	
Alkalemia (>7.45)	4 (44.4)	4 (40)	
**PO_2_** *			
Hypoxic (<65)	4 (44.4)	3 (30)	0.157
**PCO_2_** *			
Hypercapnic (>45)	2 (22.2)	3 (30)	0.288
**Hematocrit**			
Low (<34.5)	2 (22.2)	2 (20)	0.988
Normal	6 (66.7)	7 (70)	
High (>45.4)	1 (11.1)	1 (10)	
**White cell count**			
Normal	5 (55.6)	3 (30)	0.370
High	4 (44.4)	7 (70)	
**Platelets**			
Low (<150)	1 (11.1)	3 (30)	0.598
Normal	7 (77.8)	6 (60)	
High (>450)	1 (11.1)	1 (10)	
**Serum sodium**			
Low (<135)	4 (44.4)	2 (20)	0.517
Normal	3 (33.3)	5 (50)	
High (>145)	2 (22.2)	3 (30)	
**Serum potassium**			
Low (<3.5)	4 (44.4)	3 (30)	0.650
Normal	5 (55.6)	7 (70)	
High (>5.1)	0 (0)	0 (0)	
**Serum creatinine**			
Normal	2 (22.2)	4 (40)	0.628
High (>1.2)	7 (77.8)	6 (60)	
**Bilirubin**			
Normal	6 (66.7)	7 (70)	0.988
High	2 (22.2)	2 (20)	

GCS: Glasgow coma scale, ARDS: acute respiratory distress syndrome

* Mean(SD) are reported instead of n (%)

# Median (interquartile range) and Mann-Whitney U test reported for non-parametric distribution

**Table 2:** Treatment received, complications, and outcomes of influenza, and COVID-19 patients with invasive aspergillosis

	Influenza (n=9) (%)	Covid-19 (n=10) (%)	*P-value*
**Microbiology**			
Culture positive for Aspergillus	8 (88.9)	10 (100)	0.003
Serum Galactomannan positive	6 (66.7)	4 (40)	0.245
Serum Beta D-Glucan positive	5 (55.5)	3 (30)	0.762
*Aspergillus* Species			
*flavus*	3 (33.3)	8 (80)	0.025
*fumigatus*	1 (11.1)	4 (40)	0.120
*niger*	1 (11.1)	6 (60)	0.019
*terreus*	1 (11.1)	2 (20)	0.531
**Specimen for culture**			
TA	6 (66.7)	8 (80)	0.539
Sputum	2 (22.2)	2 (20)	
**Serum GM** #			
Value	0.89 (2.73) #	0.15 (2.71) #	0.278
Positive	6 (66.7)	3 (30)	
Negative	3 (33.3)	7 (70)	
**Serum BDG** #			
Value	43.63 (480)	48.21 (286)	0.762
Positive	3 (33.3)	6 (60)	
Negative	5 (55.6)	4 (40)	
**Treatment Received**			
Antifungals			
Yes	4 (44.4)	10 (100)	0.011
No	5 (55.6)	0 (0)	
**Antivirals**			
Yes	9 (100)	1 (10)	<0.001
No	0 (0)	9 (90)	
**Glucocorticoids**			
Yes	5 (55.6)	10 (100)	0.211
No	4 (44.4)	0 (0)	
**Tocilizumab**			
Yes	0 (0)	5 (50)	0.033
No	9 (100)	5 (50)	
**Convalescent plasma**			
Yes	0 (0)	3 (30)	0.211
No	9 (100)	7 (70)	
**Vasopressors**			
Yes	3 (33.3)	9 (90)	0.020
No	6 (66.7)	1 (10)	
**Intubated**			
Yes	4 (44.4)	7 (70)	0.370
No	5 (55.6)	3 (30)	
**Duration of intubation** #	0 (1) #	6.50 (12) #	0.042#
**Ventilation**			
Invasive mechanical ventilation	4 (44.4)	7 (70)	0.260
Non-invasive mechanical ventilation	7 (77.8)	8 (80)	0.906
**Complications**			
Hospital-acquired pneumonia	3 (33.3)	3 (30)	1.000
Sepsis	2 (22.2)	2 (20)	1.000
Multisystem organ failure	1 (11.1)	2 (20)	1.000
Renal failure	5 (55.6)	4 (40)	0.500
ARDS	4 (44.4)	4 (40)	0.845
**Outcome**			
Improved and discharged home	5 (55.6)	3 (30)	0.526
Expired	3 (33.3)	5 (50)	
LAMA (left against medical advice)	1 (11.1)	2 (20)	

* Mean (SD) are reported instead of n (%)

# Median (interquartile range) and Mann-Whitney U test reported for non-parametric distribution

Fever in eight (80%) and cough in eight (80%) patients were the most common presenting symptoms in patients with CAPA compared to the shortness of breath in nine (100%) patients with IAPA. The mean±SD duration of symptoms before diagnosis was 12.5±12.13 days in CAPA patients and 7.56±4.0 days in IAPA patients (*P=0.24*). Only one patient in both IAPA and CAPA groups had a history of corticosteroid intake before admission. The mean±SD duration between admission and diagnosis of aspergillosis was 5.1±2.8 and 5.0±4.7 days in CAPA and IAPA groups, respectively. Hypoxia was present in four (44.4 %) and three (30%) patients in IAPA and CAPA groups, respectively (*P=0.15*). All of the patients were found to have bilateral involvement, except for one patient who had unilateral infiltrates in the chest X-ray in the IAPA group. The mean±SD APACHE II score was 17.4±8.42 and 16.6±6.27 in patients with CAPA and IAPA, respectively (*P=0.85*). In total, nine (90%) patients required vasopressor support in the CAPA group compared to three (33%) patients in the IAPA group (*P=0.020*). Similarly, seven (70%) patients in the CAPA group required invasive mechanical ventilation compared to four (44%) in the IAPA group (*P=0.37*). The patients with aspergillosis in the CAP group did not require either mechanical ventilation or vasopressor support. All patients with IPA received voriconazole as a treatment in our study. Length of stay in hospital was longest in patients with CAPA (18.3±7.28 days) compared to those with IAPA (11.67±5.34) (*P=0.036*). Similarly, the length of stay in HDU/ICU was also longer among patients with CAPA (17±8.24) compared to those with IAPA (9.4±2.3 days) (*P=0.034*). The number of deaths in patients with IAPA and CAPA was three (33.3%) and five (50%), respectively (*P=0.526*). 

All nine patients diagnosed with IAPA had the H1N1 strain of influenza A. All patients with IPA had a positive culture for *Aspergillus* except for one patient in the IAPA group who had negative culture. *A. flavus* was the most common species found in both groups (88.9% in IAPA and 80% in CAPA patients) followed by *A. fumigatus* (33.3% in IAPA and 40% in CAPA patients). A single type of mold was found in eight (40%) patients and two species were found in 12 (60%) patients. Serum galactomannan (GM) was positive in six (66.6%) patients with IAPA and four (40%) patients with CAPA (*P=0.245*). Serum beta-D-glucan was positive in three (33.3%) patients with IAPA and in six (60%) patients with CAPA (*P=0.76*). *Acinetobacter baumannii* grew in one patient with IAPA and *Staphylococcus aureus* grew in another patient with IAPA. In patients with CAPA, *Acinetobacter baumanni* grew in four (40%), *Pseudomonas aeruginosa* in two (20 %), *Klebsiella pneumoniae* in two (20%), and *Stenotrophomonas maltophilia* in three (30%) patients.

## Discussion

Invasive pulmonary aspergillosis is now being recognized as a well-known complication of both influenza and COVID-19, despite the lack of well-established underlying immunocompromised states [ 9 , 10 ]. 

Very few studies have compared the characteristics of IAPA and CAPA. Moreover, this is the first prospective study that has compared aspergillosis in influenza, COVID-19, and CAP patients admitted within a similar time frame [ 11 - 13 ]. A total of 20 (14.2%) out of 140 patients were found to have IPA with 10 (28.5%), nine (12.8%), and one (2.8%) patient in COVID-19, influenza, and CAP groups, respectively. Florian et al. reported frequency of 24% (n=17) and 20% (n=10) for IPA in influenza and COVID-19 patients, respectively. The overall frequency of IPA was 22% (n=27) in combined influenza and COVID-19 patients in the same study which was similar to our overall frequency of 18% (n=19) [ 12 ]. In another multicenter study conducted by Bentvelsen et al., the frequency of CAPA in COVID-19 patients was reported to be 47%, which was much higher than that in the present study[ 14 ]. However, Ayalon et al. reported the frequency of CAPA in critically ill COVID-19 patients to be just 3.5%, while it was only 0.3% in overall patients with COVID-19 [ 15 ] and much lower than that in the present study and those reported by others [ 12 ]. Regional epidemiological variations in CAPA have been reported to highlight the role of an environmental load of *Aspergillus* spores, variable approach towards diagnosis, treatment of both COVID-19 and CAPA, and local genetic factors [ 16 ]. Only 3% of our CAP patients developed IPA. Consistently, this number has been reported to be 5% in another study [ 17 ]. This signifies some underlying causative factors in patients with viral pneumonia which leads to an increased frequency of IPA compared to CAP patients [ 17 ].

A previous study conducted at our institute reported a frequency of 9% for IAPA, similar to that obtained in the current study (12%). However, unlike our study, which included only severely ill patients, all admitted influenza patients were included in the study conducted by Mujahid et al. [ 18 ]. The frequency of IAPA was determined to be 44% in another study conducted in our institute on severely ill influenza patients [ 19 ]. This frequency has been reported as up to 25% in other studies [ 2 , 7 , 20 ]. A regional difference has been described in the frequency of this complication in influenza patients. This variation results from different practices of corticosteroid use, various definitions of IAPA, variable influenza vaccination rates, different levels of awareness among healthcare physicians, and possibly, environmental factors [ 7 ]. 

The study conducted by Florian et al. compared CAPA and IAPA patients and found that CAPA patients were older than IAPA patients [ 12 ]. However, the mean age of CAPA and IAPA patients in our study was similar. Moreover, the median age of CAPA patients in a study conducted by Bentvelsen et al. was 69 years, while it was 60 years in our study [ 14 ]. In the present study, a male predominance (91.4%) was found in the COVID-19 group compared to the influenza and CAP groups. Male predominance was also reported by Florian et al. and Bentvelsen et al. in both CAPA and IAPA groups [ 12 , 14 ].

*A. flavus* was the most common *Aspergillus* species found in our study in both IAPA and CAPA groups. Interestingly, this is in contrast with several other studies in other regions wherein *A. fumigatus* is the predominant species found in both influenza and COVID-19 patients [ 9 , 14 , 20 - 22 ]. Moreover, two other studies performed at our institute also showed that *A. flavus* was the predominant species in patients with aspergillosis [ 18 , 23 ]. 

In the study conducted by Lionel et al., predisposing comorbidities in patients with COVID-19 were obesity, diabetes mellitus, hypertension, and dyslipidemia. Whereas, heart failure and chronic respiratory diseases along with cirrhosis and anemia were the main predisposing comorbidities in those with influenza [ 11 ]. However, in our study, there was no significant difference between CAPA and IAPA patients with respect to underlying comorbidities. Furthermore, two other studies have shown COPD as a risk factor for the development of CAPA [ 14 , 24 ].

Renal replacement therapy was required in 37% and 50% of CAPA patients in two previous studies, respectively [ 14 , 24 ]. However, this rate was 30% (3 out of 10 ) in the present study in CAPA patients who required renal replacement therapy. In the study conducted by Florian et al., the SOFA score was lower for CAPA than IAPA patients, which was not the case in our patients [ 12 ]. A systematic review, that compared CAPA and non-CAPA patients, has also shown a statistically significant higher SOFA score for CAPA patients compared to non-CAPA patients [ 24 ]. Moreover, in the current study, the mean APACHE II score was similar in both groups. The mean APACHE II score mentioned in a study conducted by Alexander et al. was 22, while it was 16 in our influenza patients [ 2 ]. Similarly, in the study conducted by Bentvelsen et al., the median APACHE-II score was reported to be 23, while the mean APACHE II score was 17 in our CAPA patients [ 14 ]. Schauwvlieghe et al. have shown that a higher APACHE II score at the time of admission is an independent risk factor for IAPA, indicating an association between the severity of influenza and the risk of IPA development [ 2 ].

In their study, Florian et al. showed that the duration of mechanical ventilation, in-hospital death rate, and the need for vasopressors were the same between the two groups [ 12 ]. In our study, more patients in the CAPA group required vasopressors and mechanical ventilation compared to the IAPA group. Lengths of stay in hospital and HDU/ICU in our patients were longer in patients with CAPA compared to IAPA patients (*P=0.036* and *P=0.034*, respectively). However, the number of deaths in patients who had IAPA and CAPA was three (33.3%) and five (50%), respectively (*P=0.526*). Mortality in our patients with IAPA was 33%, which was lower compared to its rate (50%) in the studies performed by Alexander and Brehm [ 2 , 25 ]. Mortality due to influenza, and not IAPA, at our institute, was found to be around 16% [ 18 ]. This shows that IAPA has obviously higher mortality compared to influenza alone. In a systematic review, the rate of mortality among CAPA patients was around 48% which was similar to that found in our CAPA patients (50%) [ 26 ]. Similar mortality of 43% has been reported in another systematic review [ 24 ].

Overall, all these findings point towards more severity and poor outcomes among CAPA patients compared to IAPA patients; however, some of the differences were not statistically significant.

The majority of patients in the study conducted by Alexander et al., which showed IPA in influenza patients, had influenza A as opposed to influenza B [ 2 ]. Similarly, in our study, all nine patients in the IAPA group had the H1N1 strain of influenza A. In the study conducted by Lionel et al., the median duration between ICU admission and IPA diagnosis was six and three days in CAPA and IAPA patients, respectively [ 11 ]. Other studies have also shown that IAPA tends to develop earlier in critically ill influenza patients with a median duration of three days compared to CAPA which develops after a median of four to eight days after ICU admission [ 2 , 17 ]. However, the mean duration between admission and diagnosis of IAPA and CAPA in our patients was similar and was around five days in both groups. This relative delay in the diagnosis of IAPA in our study could be due to low suspicion of IPA in influenza patients compared to COVID-19 patients. In the study performed by Bentvelsen et al., the duration between symptom onset and CAPA diagnosis was reported to be 17 days which was higher, compared to that in the present study (12 days) [ 14 ]. 

All except one of our patients with IPA had a positive culture for *Aspergillus* in tracheal aspirate and sputum samples. BAL culture was positive in two-thirds of influenza patients with IPA in the study conducted by Alexander et al. [ 2 ]. Serum galactomannan was positive in two-thirds of our patients with IAPA which was similar to that in the study performed by Alexander et al. [ 2 ]. All 10 (100%) CAPA patients in our study had positive cultures for *Aspergillus* in respiratory secretions.

Radiology findings alone are not sufficient to diagnose CAPA. However, multiple nodules or lung cavitation should prompt further investigation for IPA. Although BAL and lung biopsy specimens are the samples of choice for the diagnosis of IPA, bronchoscopy has very little role in the diagnosis of CAPA due to the transmission risk of COVID-19 infection through aerosol generation. Therefore, diagnosis of a proven CAPA is very difficult, and there is a need for defining more practical and feasible diagnostic criteria for CAPA. These may include high volume culture and *Aspergillus* antigen on tracheal aspirates. However, these criteria should not overdiagnose cases of IPA which may otherwise lead to undue exposure to potentially hepatotoxic and nephrotoxic antifungal medications for relatively long durations. This situation has become more complex with the low diagnostic yield of serum galactomannan in CAPA patients. This should be weighed against the fact that *Aspergillus* should not be disregarded merely as a colonizer in these populations of patients due to substantially higher morbidity and mortality associated with IAPA and CAPA [ 1 , 4 , 27 ]. 

Our study has some limitations. This is a single-center study with a limited sample size, which does not allow for multivariate analysis. None of the patients in our study underwent bronchoscopy which renders IPA diagnosis very challenging. Our data did not include any cases of proven invasive aspergillosis since getting a tissue-based diagnosis is very difficult in critically ill patients who rarely undergo invasive procedures. Eventually, there is a need for similar studies with larger sample sizes to further understand this disease entity in COVID-19 and influenza patients.

## Conclusion

A higher proportion of patients with COVID-19 developed invasive aspergillosis compared to influenza. Although the mortality rate in CAPA and IAPA patients was comparable, CAPA patients had a significantly longer length of stay in hospital and critical care units with a higher requirement for vasopressors and mechanical ventilation.

## Acknowledgments

We would like to acknowledge the statistical support provided by Dr. Saadbin Zafar for this study.

## Authors’ contribution

S.A.A. conceived the study, acquired funding, wrote the proposal, and conducted the study, literature search, and manuscript writing. K.J. wrote, reviewed, and edited the manuscript, did the literature search, and supervised the study. J.F. reviewed and edited the manuscript and formal analysis. A.F.S. extracted the data, took consent from patients and families, performed a literature search, and reviewed the manuscript. H.N. performed the literature search and formal analysis. S.A. conducted a data analysis plan and formal analysis. A.A. extracted the data, took consent from patients and families, undertook the literature search, and reviewed the manuscript. M.I. wrote, reviewed, and edited the manuscript, conceived the study, devised methodology, supervised the study, and undertook manuscript writing and editing. All authors contributed to the final version of the manuscript and approved it for publication. 

## Conflicts of interest

The authors declare that they have no conflicts of interest to express. The abstract of this study has been submitted to TIMM (Trends in Medical Mycology) 2021 Meeting.

## Financial disclosure

This research project received a grant from the Seed Money Program for Research Development of The Aga Khan University Hospital in Karachi, Pakistan (Grant No. PF119/1118).
